# Teaming-up nurses with ophthalmologists to expand the reach of eye care in a middle-income country: Validation of health data acquisition by nursing staff in a telemedicine strategy

**DOI:** 10.1371/journal.pone.0260594

**Published:** 2021-11-30

**Authors:** Cassia Garcia Moraes Pagano, Tais de Campos Moreira, Daniel Sganzerla, Ana Maria Frölich Matzenbacher, Amanda Gomes Faria, Lucas Matturro, Felipe Cezar Cabral, Dimitris Rucks Varvaki Rados, Anelise Decavata Szortyka, Maicon Falavigna, Maria Eulalia Vinadé Chagas, Erno Harzheim, Marcelo Gonçalves, Roberto Umpierre, Aline Lutz de Araujo

**Affiliations:** 1 Hospital Moinhos de Vento, Porto Alegre, RS, Brazil; 2 Programa de Pós Graduação em Epidemiologia, Universidade Federal do Rio Grande do Sul, Porto Alegre, RS, Brazil; 3 Núcleo de Telessaúde, Universidade Federal do Rio Grande do Sul, Porto Alegre, RS, Brazil; 4 Departamento de Oftalmologia e Ciências Visuais, Universidade Federal de São Paulo, São Paulo, SP, Brazil; University of Warmia, POLAND

## Abstract

Telemedicine can be used to conduct ophthalmological assessment of patients, facilitating patient access to specialist care. Since the teleophthalmology models require data collection support from other health professionals, the purpose of our study was to assess agreement between the nursing technician and the ophthalmologist in acquisition of health parameters that can be used for remote analysis as part of a telemedicine strategy. A cross-sectional study was conducted with 140 patients referred to an ophthalmological telediagnosis center by primary healthcare doctors. The health parameters evaluated were visual acuity (VA), objective ophthalmic measures acquired by autorefraction, keratometry, and intraocular pressure (IOP). Bland-Altman plots were used to analyze agreement between the nursing technician and the ophthalmologist. The Bland-Altman analysis showed a mean bias equal to zero for the VA measurements [95%-LoA: -0.25–0.25], 0.01 [95%-LoA: -0.86–0.88] for spherical equivalent (M), -0.08 [95%-LoA: -1.1–0.95] for keratometry (K) and -0.23 [95%-LoA: -4.4–4.00] for IOP. The measures had a high linear correlation (R [95%CI]: 0.87 [0.82–0.91]; 0.97 [0.96–0.98]; 0.96 [0.95–0.97] and 0.88 [0.84–0.91] respectively). The results observed demonstrate that remote ophthalmological data collection by adequately trained health professionals is viable. This confirms the utility and safety of these solutions for scenarios in which access to ophthalmologists is limited.

## Introduction

Visual impairment is a serious problem in many different populations [1]. According to the World Health Organization (WHO), it was estimated that in 2015 there would be 36 million blind people in the world and millions more people with visual impairment lacking adequate treatment [2]. In Tropical Latin America, which includes Brazil, 1.64% of the population have visual problems considered severe or moderate [3]. The WHO developed a global action plan to reduce avoidable visual impairment and offer treatment to people with visual impairment. One of the action plan’s goals was to increase investment in scientific research, with the objective of developing cost-effective, evidence-based diagnostic techniques and interventions with adequate strategies for eye health care, particularly for mid and low-income populations [2].

Employing telemedicine in ophthalmology is one way of facilitating patient access to specialist care, particularly for those in localities where there is a dearth of ophthalmologists, reducing travel costs, time off work, and waiting times for consultations [4]. This technology offers real-time interaction between doctors and patients and enables examinations to be conducted at a distance [5]. Telemedicine can be used to conduct ophthalmological assessment of patients, facilitating screening for diseases such as diabetic retinopathy and glaucoma [6] and enabling correction of refractive errors [7].

Teleophthalmology models currently in use require data collection support from other health professionals, such as technology professionals, nurses, or nursing technicians. These trained professionals provide in-person support for collection of parameters, which may be conducted under ophthalmologist supervision, or unsupervised, and may be synchronous or asynchronous [7–9].

An important challenge to be overcome in implementing teleophthalmology is to guarantee the quality of the data collected by these health professionals, which is generally based on training of human resources, standardization of routines, and regular assessments of the performance required to provide the reliable data needed for distance diagnosis [8, 10]. In this scenario, assessment of the fundamental parameters measured by the professionals involved in telediagnosis is extremely relevant to achieving development and ensuring the reliability of telemedicine.

The coronavirus disease 2019 pandemic illustrates the increasingly important role of telemedicine as a method of clinician-patient interaction. In this scenario, investigating interobserver agreement between specialist doctors and support professionals when acquiring health data for transmission and remote interpretation is fundamental for the validity of remote diagnosis. The objective of this study was to assess agreement between the nursing technician and the ophthalmologist in acquisition of health parameters that can be used for remote analysis as part of a telemedicine strategy. The health parameters evaluated in this study were visual acuity and objective ophthalmic measures acquired by autorefraction, keratometry, and air-jet intraocular pressure measurement.

## Methods

A cross-sectional study was conducted during the months from October to December of 2017 at a teleophthalmology center in the city of Porto Alegre, Brazil. A total of 140 patients with ophthalmological complaints who had been referred to the ophthalmological telediagnosis center by primary care (PC) doctors were enrolled consecutively on the study. The teleophthalmology center (TeleOftalmo Project) consists of two remote examination rooms where visual acuity tests and automated refractometry and keratometry acquisition are conducted, photos are taken of the anterior segment of the eye under a slit-lamp, and retinography and non-contact air-jet tonometry are performed by a nursing team (nursing technicians and nurses) with support from ophthalmologist doctors who are responsible for overseeing and supervising the examinations via video links. The off-site ophthalmologist remotely evaluates the images and data simultaneously or immediately after its collection by the nurse technicians.

The objective of the ophthalmological telediagnosis model adopted is to diagnose eye conditions that are prevalent among patients cared for on the Brazilian National Health Service (SUS—Sistema Único de Saúde). Doctors working in PC refer patients with nonspecific low visual acuity, refractive errors, strabismus, eyelid lesions, conjunctival lesions, and cataracts without prior indication for surgery and also diabetic patients for diabetic retinopathy screening. Patients in need of emergency treatment or with indications for medical or surgical treatment (e.g. laser, intraocular corticoids) were not eligible for the study [11].

Data collection was conducted independently, in two remote examination rooms equally equipped with an ophthalmology specialist experienced in ambulatory consultations and by nursing technicians (NT) who had been trained in advance to collect the data involved. The NT had undertaken a training program with ophthalmological doctors, 10 hours of which were dedicated to theoretical-practical training on acquisition of examinations conducted at the TeleOftalmo Project [11] and another 14 hours of which were dedicated to practical training with the equipment used. At the time of data collection, the NT had been working at the project for 4 months and had had no previous experience with teleophthalmology.

Patients were randomized to be examined first by the NT or by an ophthalmologist. The randomization list was generated electronically and a nurse supervisor who did not take part in the examinations was responsible for patient allocation.

All examiners followed a standardized assessment protocol used for consultations at the ophthalmological telediagnosis center and data were collected using standardized and pretested forms. After each patient had undergone all of the examinations, the NT recorded the results on an on-line platform, used to store patients’ registration data, in accordance with the normal flow of care. The ophthalmology specialist recorded the same information for each patient on an identical electronic form. Both sets of data collection were performed on the same day, in random order, and in sequential time. On average, patients were seen by the nurse technicians and by the ophthalmologist between 30 minutes and 1 hour apart.

The following data were collected: visual acuity (VA) without correction, using the modified Bailey-Lovie chart, refractomety and automated keratometry (Visuref 100®, Zeiss, Germany), and intraocular pressure (IOP), measured by non-contact air jet tonometry (Visuplan 500®, Zeiss, Germany).

Visual acuity was measured one eye at a time, covering the left eye (LE) when testing the right eye (RE) and the opposite when testing the LE, with the VA chart placed 4 meters away from the patient. During each test, the patient read the letters shown on each line out loud, or indicated the position of the tumbling E in the case of illiterate patients, starting with the line of the largest optotypes. For each measurement, the smallest line on which at least 3 of the 5 optotypes were correctly identified was noted. The measure was recorded as a Snellen fraction together with the number of optotypes not identified on the same line (for example, 20/25–2) or identified on the following line (for example, 20/25 +1). The autorefraction, keratometry, and IOP measurements were recorded as the mean of three automated measurements performed by the machines. Examinations were conducted one eye at a time, starting with the RE.

For the purposes of analysis and interpretation of results, the following cutoffs were predefined, as specified in Table 1:

- Normative values for parameters: The usual cutoff points employed in clinical practice were adopted, for analysis of the capacity of each test to detect suspected or abnormal cases. The use of cutoff points in this study was based on a theoretical scenario in which each test, in isolation, would be indicative of a given clinical condition or suspicion of that condition (for example, IOP exceeding 21mmHg as indicative of ocular hypertension, glaucoma, or suspected glaucoma). For visual acuity, a cutoff point of 20/40 was adopted, in line with the new WHO classification of visual impairment (ICD-11).
- Expected variability: For the VA measurement, variability between repeated measures was expected to be in line with a previous test-retest study in the literature [12]. For autorefraction, automated keratometry and IOP, the assumed variability was that indicated by the manufacturer of the equipment, if available, or if not available, data from previous studies in the literature using similar equipment were used [13–15].

**Table 1:** Limits of normality and expected variability for the ophthalmological parameters assessed.

Ophthalmological parameters	Limit of normality	Variability expected
Visual acuity	20/40 or 0.3 logMAR	1 line or 0.1 logMAR
Autorefraction	< 0.5 diopters	0.5 diopters
Keratometry	< 48 diopters	0.32 diopters
Intraocular pressure	≤ 21mmHg	4 mmHg

### Analysis of the data

Since we have four different measures, with same importance for study objectives, with uncertainty for dispersion parameters, the sample size estimate was based on effect size. Assuming a design effect (DEFF) of 1.7 [16], the study would need a sample size of 262 pairs (131 patients) to achieve 80% power and a 5% significance level (two tailed) to detect a standardized mean difference of 0.23 (Cohen’s d) between pairs, that is considered a small to non-important effect size in health sciences [17]. Allowing for possible losses, a sample of 140 patients would be sufficient.

Continuous variables were expressed as means and standard deviations, or medians with interquartile ranges, while categorical variables were expressed as absolute and relative frequencies, with cumulative frequencies when appropriate. To perform the statistical analysis comparing the two assessments, the VA measurements were converted into the logarithm of the minimum resolution angle (logMAR), which corresponds to a continuous measure of VA with a linear scale, suitable for quantitative comparison of the mean VA. Visual acuities that were not covered by the values contained in the table employed (for example, finger counting or light/dark perception) were excluded from the analysis. The components of refraction measurement (sphere, cylinder, and axis) were converted to power vectors using the method described by Thibos and Horner [18], to obtain the independent parameters M, J0, and J45, in diopters (D). Cohen’s Kappa was calculated to measure the inter-rater reliability between the nursing technician and the ophthalmologist measurements in categorical variables. Bland-Altman plots were used to analyze agreement between the nursing technician and the ophthalmologist, in addition to scatter plots of comparison of assessments. The differences between measures taken by the nursing technicians and the ophthalmologists were assessed using mixed generalized linear models, to consider the dependence between the measurements and eyes. Sensitivity, specificity, Cohen’s Kappa and 95% confidence intervals [95%CI] were calculated for VA and IOP, using the ophthalmologists’ results as the reference standard. For visual acuity, a cutoff point of 20/40 was adopted, in line with the new WHO classification of visual impairment (ICD-11) and a cutoff point of ≥ 21mmHg as indicative of ocular hypertension, glaucoma, or suspected glaucoma.

Statistical analyses were conducted using R Core Team statistical software [19]. Informed consent was obtained from participants after explanation of the nature of the study and its possible consequences. The study was approved by the Research Ethics Committee at the Hospital de Clínicas de Porto Alegre (CAAE 64499316.1.0000.5327, consolidated opinion number 2.757.077). A printed consent form was applied and the participants signed in 2 copies, one kept by the researchers and the other delivered to the participants. The study did not include underage participants.

## Results

A total of 140 patients were enrolled on the study, with a median age of 53 years [IQR: 39.0–65.0], 67.1% (94/140) of whom were female and 69.3% (97/140) of whom were white. A total of 254 visual acuity measurements were taken, counting those done by the doctors and the technicians, with 270 keratometry measurements, and 278 intraocular pressure measurements.

Twenty-six of the visual acuity measurements were off the acuity chart, because vision was worse than the lower limit (visual acuity measurements using finger count or light perception), and were therefore excluded from the analysis. Twenty-six, 10, and 2 autorefraction, keratometry, and intraocular pressure measurements, respectively, could not be collected because of equipment limitations (for example, keratometry “out of range”) or because of anatomic ocular abnormalities that prevented measurement (for example, an opaque cornea).

### Agreement between visual acuity assessments

Mean VA measured by the doctor was 0.22 logMAR [95%CI: 0.00–0.50]. The difference between the measurements made by technicians and the doctors (0.00 [95%CI]: 0.06–0.08) was not statistically significant (p = 0.495).

**Fig 1:**
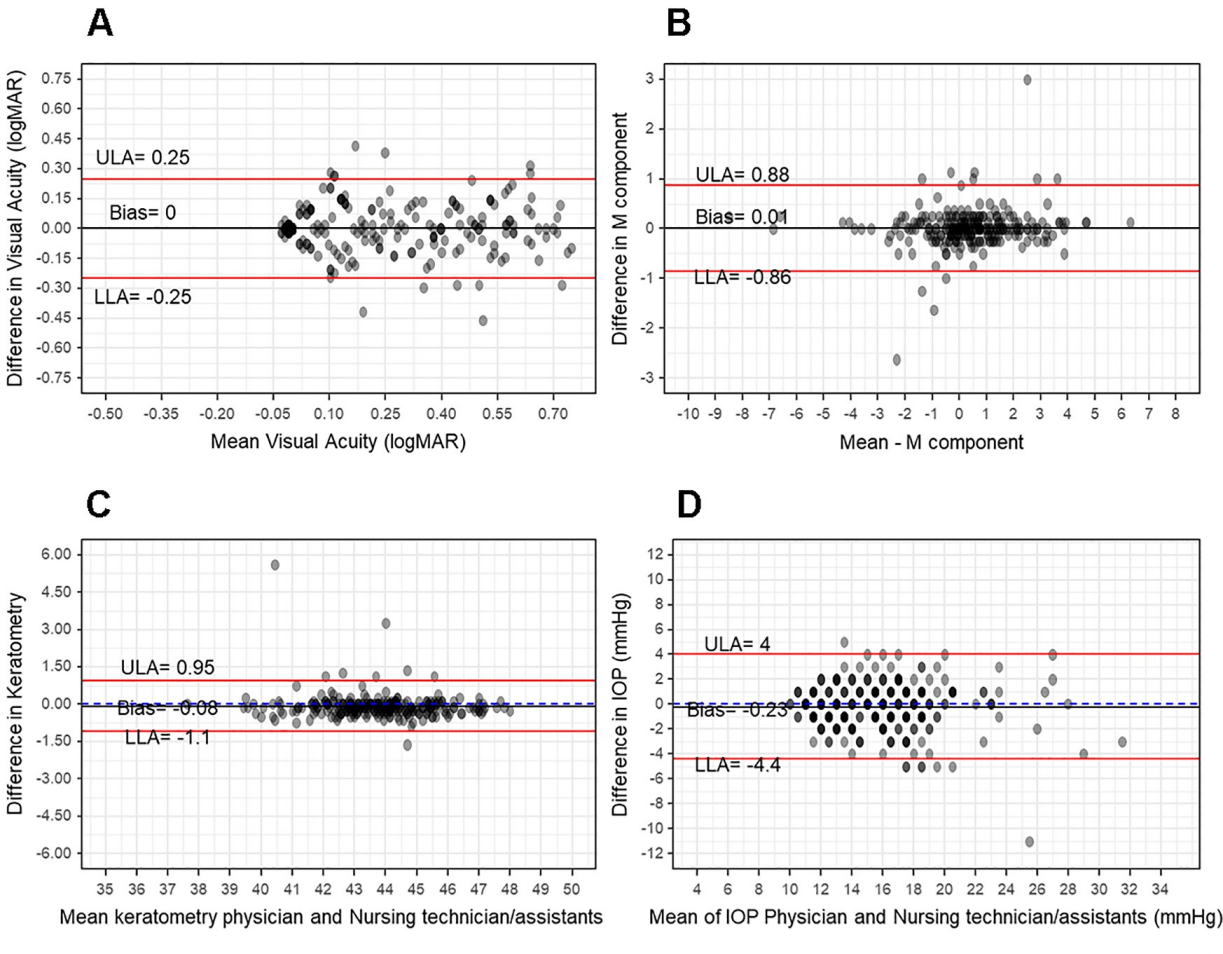
Bland-Altman plots comparing assessments made by doctors and nursing technicians for: A) Visual acuity, in logMAR; B) Autorefraction spherical equivalent (M), in diopters; C) Keratometry, in diopters; and D) Intraocular pressure, in mmHg.

**Fig 2:**
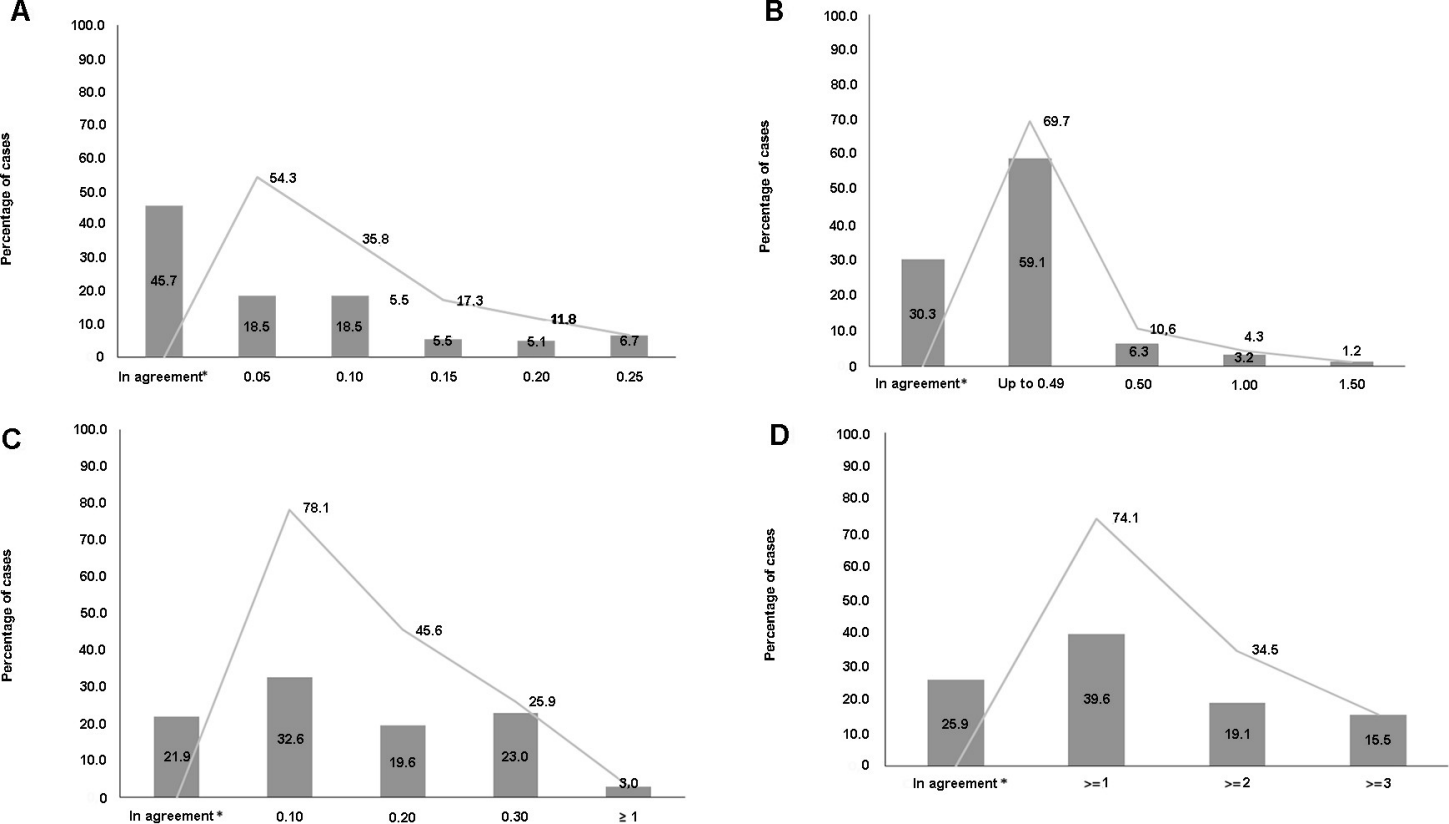
Percentage disagreement between doctor and nursing technicians for: A) Visual acuity, in logMAR; B) Autorefraction spherical equivalent (M), in diopters; C) Keratometry, in diopters; and D) Intraocular pressure, in mmHg.

The Bland-Altman plot (Fig 1A) showed a mean bias equal to zero for the VA measurements taken by the doctors and the nursing technicians, and the limits of agreement showed that the differences between the two measures were less than -0.25 and 0.25 logMAR. The measures had a high linear correlation (R [95%CI]: 0.91 [0.88–0.93]; p<0.001). A total of 254 eyes were assessed, of which the VA measured for 116 pairs (45.7%) were perfectly in agreement, while disagreement a maximum of 0.10 logMAR for 94 pairs (37.0%) (Fig 2A). Therefore, 210 pairs (82.7%) exhibited agreement within the expected inter-test variability (up to 0.10 logMAR). Considering the preestablished criterion for low VA (>0.3 logMAR), 108 eyes (42.5%) were detected with low VA according to the doctors’ measurements and there was disagreement for 20 eyes (7.87%). Agreement for detection of low acuity was 92.0% ([95%CI]: 88.0–95.0), with a kappa of 0.86 ([95%CI]: 0.77–0.95). The diagnostic properties for VA assessment are shown in Table 2.

**Table 2:** Diagnostic properties of assessment of visual acuity by doctors (gold standard) and nursing technicians.

	Ophthalmology specialists		
Technicians	Visual impairment	VA normal	Total
Visual impairment	98	10	108
VA normal	10	136	146
Total	108	146	254

Sensitivity: 0.91 ([95%CI]: 0.84–0.95); Specificity: 0.93 ([95%CI]: 0.88–0.97); Positive predictive value: 0.91 ([95%CI]:0.84–0.95).

Negative predictive value: 0.93 ([95%CI]: 0.88–0.97).

Visual impairment defined as acuity exceeding 0.3 LogMAR.

VA, visual acuity.

Considering the order of tests, patients’ performance was better in the second VA, irrespective of whether the measurement was taken by the technicians or the doctors (data shown in Supporting Information). No differences were observed in agreement related to laterality (RE or LE).

### Agreement in autorefraction assessments

The difference between means for technicians and doctors for the autorefraction spherical equivalent (0.00 [95%CI]: -0.13–0.13) was not statistically significant (p = 0.690).

The Bland-Altman plot (Fig 1B) illustrates the data for spherical equivalent (M) results. Bias was 0.01D between doctors and nursing technicians and the limits of agreement indicate that the differences between the two measures fall within the range of -0.86 to 0.88D. The correlation between measurements was 0.97 ([95%CI]: 0.96–0.98; p<0.001).

The spherical equivalent M results were in agreement for 77 assessments (30.3%), while 166 assessments (65.4%) had up to 0.5 D disagreement (Fig 2B). No differences in the agreement assessment were observed in relation to laterality or order of assessment (doctor first or technician first).

### Agreement in surface curvature of the cornea (keratometry) assessments

The mean doctor-assessed keratometry result (K) was 43.8D ([95%CI]: 42.8–45.0). The difference between means for technicians and doctors (-0.13 [95%CI]: -0.25–0.00) was significant (p<0.001), but was not clinically relevant.

The Bland-Altman plot (Fig 1C) illustrates the data for keratometry (K) results in diopters (D). Bias was -0.08D between doctors and nursing technicians and the limits of agreement indicate that the differences between the two measures fall within the range of -1.11 to 0.95D. The correlation between measurements was 0.96 ([95%CI]: 0.95–0.97; p<0.001).

For the keratometry assessments, results for 59 pairs of eyes (21.9%) were in agreement and results for 203 pairs of eyes (75.2%) disagreed by less than 1D (Fig 2C). The test’s diagnostic properties could not be evaluated because just 1 participant had a value above the limit of normality (>48D) according to measurements taken by the doctor and none were outside of the limits of normality according to the nursing technician. No differences in the agreement assessment were observed in relation to left or right eyes or order of assessment.

### Agreement in intraocular pressure measurements

The mean ophthalmologist-assessed IOP of patients was 15.0 mmHg ([95%CI]: 13.0–18.0). The Bland-Altman plot (Fig 1D) illustrates the data for IOP in mmHg. Bias between doctors and nursing technicians was -0.23 mmHg and the limits of agreement indicate that the differences between the two measures fall within the range of -4.44 to 4.00 mmHg. The correlation between measurements was 0.88 ([95%CI]: 0.84–0.91; p<0.001).

The difference between means for technicians and doctors (0.00 [95%CI]: -1.00–1.00) was not statistically significant (p = 0.085). A total of 72 IOP measurements (25.9%) were in agreement and 163 (58.6%) disagreed by up to 2 mmHg (Fig 2D).

Considering the outcome suspected glaucoma (IOP ≥ 21 mmHg), 20 patients had IOP ≥ 21 mmHg according to the doctors’ assessment, compared to 17 patients according to the nursing technicians’ results, with diagnostic agreement of 98.0% ([95%CI]: 96.0–99.0) and kappa of 0.86 ([95%CI]:0.73–0.98). The diagnostic properties for assessment of suspected glaucoma are shown in Table 3.

**Table 3:** Diagnostic properties of assessment of intraocular pressure measured by doctor (gold standard) and nursing technicians.

	Ophthalmology specialists		
Nursing technicians	IOP > 21mmHg	IOP ≤ 21mmHg	Total
IOP > 21mmHg	16	1	17
IOP ≤ 21mmHg	4	257	261
Total	20	258	278

Sensitivity: 0.80 ([95%CI]:0.56–0.94); Specificity: 1.00 ([95%CI]: 0.98–1.00); Positive predictive value: 0.94 ([95%CI]:0.71–1.00)

Negative predictive value: 0.98 ([95%CI]: 0.96–1.00).

No differences in the agreement assessment were observed in relation to left or right eyes or order of assessment (S1 Fig).

Correlation plots for nursing technicians against doctors’ assessments for all measures analyzed (S2 Fig) and Bland-Altman plots for LE against RE (S3 Fig) are provided in the Supporting information.

## Discussion

Our results show that there were small discrepancies between the data collected by the doctors and by technicians. However, these discrepancies did not have major clinical relevance that could impact the ophthalmologist’s decision on the patient’s diagnosis. These findings provide evidence of the safety of teleophthalmology strategies with in-person support from nursing technicians.

Some methodological aspects of our study merit more in-depth discussion. Part of the variability in visual acuity assessments can be explained by the inherent effects of repeated measures in patients and would also be observed in repeated assessments conducted by a single professional.

In relation to visual acuity, patients may memorize the letters shown on the Snellen chart because of their repeated use during ophthalmological examinations [20]. This explains the fact that patients performed better during the second administration and, as a result, analysis of agreement between measures revealed greater variability, albeit still acceptable. The acuity charts used for data collection displayed the same optotypes, which is a limitation of the study that could have facilitated memorization by the patients. Additionally, measurement of acuity requires adequate training, experience with data collection, and support from qualified professionals to be conducted satisfactorily [21]. The nursing technicians who took measurements at the project only had 4 months’ experience and this may also have been an important factor in some of the measurement discrepancies.

The autorefraction measure, expressed as the spherical equivalent M, exhibited satisfactory agreement in our study. The eye’s refraction is subject to physical accommodation fluctuations, with frequency of 1 to 5 Hz and amplitude of approximately ± 0.1 to ± 0.25 D of ideal accommodation [22, 23] Subjective refraction is considered the gold-standard method for determination of the refractive status of the eyes, because it takes into consideration important factors not accounted for by autorefractors, such as neural processes and binocular equilibrium, although it too exhibits interobserver variability [24]. Because of this variability, refraction errors are not normally assessed to precision exceeding ± 0.25 D for smaller refraction errors or ± 0.50 D for larger refraction errors [25].

The keratometry measurements exhibited good agreement in the data from our study, but the spectrum of patients was restricted to healthy patients, so the data do not reveal how good performance would be in examinations of patients with keratoconus or other diseases of the cornea. Since it is a disease that does not reveal symptoms or clinical signs, it is extremely important to diagnose keratoconus during the initial stages of the disease [26]. Measurements of refraction and visual acuity are less sensitive for keratoconus than topography, but changes in these measurements are nevertheless important signs of the progression of this disease [27]. Our results cannot therefore be generalized to patients with suspected keratoconus.

The variability of examinations such as intraocular pressure measurement is dependent on minor variations such as patient positioning and breathing. In our study, agreement between tonometry measurements taken by nursing technicians and ophthalmology specialists was acceptable, but discrepancies did affect the classification of diagnostic suspicion for some patients. A number of important factors can interfere with this measure. In this study, patients were examined using Zeiss non-contact tonometers, with an expected variability of up to 4 mmHg, according to the manufacturer [28] and since the variability between professionals was within this limit, we can infer that the nursing technicians’ assessment did not exhibit variability, considering expected variability. Non-contact tonometers (NCTs) have the advantage of pushing back the cornea with an air jet, reducing the possible risk of cross-infection, but are not always precise [29, 30]. Repeated readings with contact tonometers are more reproducible than other automated measurements [29] and are the gold standard for IOP measurement. Kotecha et al. [31] published studies showing that ophthalmologist doctors achieve better agreement in IOP measurements using contact tonometers and nursing technicians have better agreement with non-contact tonometers. Other variables can also interfere with IOP measures, including astigmatism, corneal thickness, biomechanics, accommodation, breathing, heart rate, and daily variation, resulting in a maximum clinically acceptable variation of ± 3.0 mmHg [32].

Although the study has some limitations, such as the nursing technicians’ length of experience with assessment of ophthalmological parameters (4 months), use of air-jet tonometry measurements only, and use of acuity charts showing the same optotypes, the results observed show that assessment by nursing technicians exhibits acceptable agreement.

One relevant aspect of the setting in which this study was conducted is that there is no specific professional category for ophthalmological assistants in Brazil and so very often nursing technicians are trained to perform this role, as is the case at the service where the study was conducted. Similar to Brazil, other countries in Latin America don’t have standard professionals who can assist ophthalmologists. In Peru, for example, there are few certified ophthalmological nurses, the majority of them being trained in their work center only. In Honduras, healthcare professionals who have a role assisting in ophthalmologic procedures do not have formal training. The optometrist is only formally recognized in Peru, Honduras and Mexico. Meanwhile, countries such as Chile and Uruguay have ophthalmological technicians who collect refraction data [33].

It is known that continuous training and continuing education lead to establishment of standards for the quality of the assessments conducted by these professionals. In other countries, ophthalmology assistant professionals have helped in screening and follow-on ophthalmological diseases such as glaucoma and diabetic retinopathy in primary care [34–36].

Even though some studies have shown good accuracy in telemedicine diagnosis in the ophthalmology field [37–39]. The evaluation of the data necessary to perform a synchronous remote diagnosis, collected by technicians who are not specialists in ophthalmology, is important in order to validate the care provided by a non-medical professional for ophthalmological diagnosis by telemedicine.

The results found demonstrate that actions employing distance ophthalmological data collection by adequately trained health professionals with secondary or technical education are feasible options for providing care to populations that do not have access to specialist services. With support from adequately trained health professionals, ophthalmological healthcare provided via telemedicine enables access to eye health to be extended to populations with limited access to traditional services.

## Supporting information

S1 Fig Graphs illustrating correlation between assessments by nursing technicians and doctors: A. Visual acuity, in logMAR; B. Spherical equivalent M, for refraction in diopters; C. Keratometry, in diopters; D. Intraocular pressure, in mmHg.
(PDF)

S2 Fig Graph illustrating correlation and discrepancies between measures by order of examination: A) Visual acuity; B) Spherical equivalent M; C) Keratometry; D) Intraocular pressure.
(PDF)

S3 Fig Assessment correlation graphs and Bland-Altman plots comparing assessments by doctors and nursing technicians for: A) Visual acuity for right eye; B) Visual acuity for left eye; C) Spherical equivalent M for right eye; D) Spherical equivalent M for left eye; E) Keratometry for right eye; F) Keratometry for left eye. G) Intraocular pressure for right eye; H) Intraocular pressure for left eye.
(PDF)

S1 Table Database acquisition by nursing staff. (XLSX)

## References

[1] Bourne RRA, Flaxman SR, Braithwaite T, et al. Magnitude, temporal trends, and projections of the global prevalence of blindness and distance and near vision impairment: a systematic review and meta-analysis. Lancet Glob Health. 2017;5:e888–e897. doi: 10.1016/S2214-109X(17)30293-0 28779882

[2] World Health Organization (WHO). Draft action plan for the prevention of avoidable blindness and visual impairment 2014–2019. Universal eye health: a global action plan 2014–2019. 2013. https://www.who.int/blindness/AP2014_19_English.pdf?ua=1. Accessed 4 Nov 2020.

[3] Leasher JL, Braithwaite T, Furtado JM, et al. Prevalence and causes of vision loss in Latin America and the Caribbean in 2015: magnitude, temporal trends and projections. Br J Ophthalmol. 2019;103:885–93. doi: 10.1136/bjophthalmol-2017-311746 30209083

[4] Sapru S, Berktold J, Crews JE, et al. Applying RE-AIM to evaluate two community-based programs designed to improve access to eye care for those at high-risk for glaucoma. Eval Program Plann. 2017;65:40–6. doi: 10.1016/j.evalprogplan.2017.06.006 28689028

[5] Kane CK, Gillis K. The use of telemedicine by physicians: still the exception rather than the rule. Health Aff (Millwood). 2018;37:1923–30. 30633670 10.1377/hlthaff.2018.05077

[6] Sommer AC, Blumenthal EZ. Telemedicine in ophthalmology in view of the emerging COVID-19 outbreak. Graefe’s Arch Clin Exp Ophthalmol. 2020;258: 2341–2352. doi: 10.1007/s00417-020-04879-2 32813110 PMC7436071

[7] Suram V, Addepalli UK, Krishnaiah S, et al. Accuracy of vision technicians in screening ocular pathology at rural vision centres of southern India. Clin Exp Optom. 2016;99:183–7. doi: 10.1111/cxo.12345 27012692

[8] Paudel P, Cronjé S, O’Connor PM, et al. Clinical competency of 1-year trained vision technicians in Andhra Pradesh, India. Ophthalmic Epidemiol. 2015;22:409–16. doi: 10.3109/09286586.2015.1082605 26653263

[9] Hark LA, Katz LJ, Myers JS, et al. Philadelphia telemedicine glaucoma detection and follow-up study: methods and screening results. Am J Ophthalmol. 2017;181:114–24. doi: 10.1016/j.ajo.2017.06.024 28673747

[10] Safi S, Ahmadieh H, Katibeh M, et al. Modeling a telemedicine screening program for diabetic retinopathy in Iran and implementing a pilot project in Tehran suburb. J Ophthalmol. 2019;2019:2073679. doi: 10.1155/2019/2073679 30949361 PMC6425400

[11] De Araujo AL, Moreira TC, Rados DRV, et al. The use of telemedicine to support Brazilian primary care physicians in managing eye conditions: The TeleOftalmo Project. PLoS One. 2020;15:e0231034. doi: 10.1371/journal.pone.0231034 32240268 PMC7117761

[12] Plainis S, Tzatzala P, Orphanos Y, et al. A modified ETDRS visual acuity chart for European-wide use. Optom Vis Sci. 2007;84:647–53. doi: 10.1097/OPX.0b013e3180dc9a60 17632314

[13] Hua Y, Xu Z, Qiu W, et al. Precision (repeatability and reproducibility) and agreement of corneal power measurements obtained by Topcon KR-1W and iTrace. PLoS One. 2016;11:e0147086. doi: 10.1371/journal.pone.0147086 26752059 PMC4709181

[14] Elliott M, Simpson T, Richter D, et al. Repeatability and comparability of automated keratometry: the Nikon NRK-8000, the Nidek KM-800 and the Bausch and Lomb keratometer. Ophthalmic Physiol Opt. 1998;18:285–93. 9829116

[15] Shirayama M, Wang L, Weikert MP, et al. Comparison of corneal powers obtained from 4 different devices. Am J Ophthalmol. 2009;148:528–35. doi: 10.1016/j.ajo.2009.04.028 19541287

[16] Murray IJ, Hassanali B, Carden D. Macular pigment in ophthalmic practice; a survey. Graefe’s Arch Clin Exp Ophthalmol = Albr von Graefes Arch fur Klin und Exp Ophthalmol. 2013;251: 2355–2362. doi: 10.1007/s00417-013-2430-4 23912797

[17] Kazis LE, Anderson JJ, Meenan RF. Effect sizes for interpreting changes in health status. Med Care. 1989 Mar;27(3 Suppl):S178–89. doi: 10.1097/00005650-198903001-00015 2646488

[18] Thibos LN, Horner D. Power vector analysis of the optical outcome of refractive surgery. J Cataract Refract Surg. 2001;27:80–5. doi: 10.1016/s0886-3350(00)00797-5 11165859

[19] R Foundation for Statistical Computing. R Core Team. A language and environment for statistical computing. 2020. https://www.R-project.org/. Accessed 04 Nov 2020.

[20] Chen FK, Agelis LE, Peh KK, et al. Factors contributing to discrepancy between visual acuity fractions derived from a Snellen chart and letter scores on the early treatment diabetic retinopathy study chart. Asia Pac J Ophthalmol (Phila). 2014;3:277–85. doi: 10.1097/APO.0000000000000007 26107914

[21] Shah PR, Yohendran J, Hunyor AP, et al. Uveal effusion: clinical features, management, and visual outcomes in a retrospective case series. J Glaucoma. 2016;25:e329–35. doi: 10.1097/IJG.0000000000000329 26550979

[22] Grein HJ, Schmidt O, Ritsche A. [Reproducibility of subjective refraction measurement]. Ophthalmologe. 2014;111:1057–64. doi: 10.1007/s00347-014-3064-6 25412602

[23] Hervella L, Villegas EA, Prieto PM, et al.Assessment of subjective refraction with a clinical adaptive optics visual simulator. J Cataract Refract Surg. 2019;45:87–93. doi: 10.1016/j.jcrs.2018.08.022 30309774 PMC6320260

[24] Pujol J, Ondategui-Parra JC, Badiella L, et al. Spherical subjective refraction with a novel 3D virtual reality based system. J Optom. 2017;10:43–51. doi: 10.1016/j.optom.2015.12.005 26856962 PMC5219830

[25] Smith G. Refraction and visual acuity measurements: what are their measurement uncertainties? Clin Exp Optom. 2006;89:66–72. doi: 10.1111/j.1444-0938.2006.00022.x 16494608

[26] Hashemi H, Beiranvand A, Yekta A, et al. Pentacam top indices for diagnosing subclinical and definite keratoconus. J Curr Ophthalmol. 2016;28:21–6. doi: 10.1016/j.joco.2016.01.009 27239598 PMC4881219

[27] Ferdi AC, Nguyen V, Gore DM, et al. Keratoconus natural progression: a systematic review and meta-analysis of 11 529 eyes. Ophthalmology. 2019;126:935–45. doi: 10.1016/j.ophtha.2019.02.029 30858022

[28] Guia de instruções de uso Visuplan 500 Tonômetro sem contato, 2017 Carl Zeiss Meditec AG, Jena.

[29] Tonnu PA, Ho T, Sharma K, et al. A comparison of four methods of tonometry: method agreement and interobserver variability. Br J Ophthalmol. 2005;89:847–50. doi: 10.1136/bjo.2004.056614 15965164 PMC1772716

[30] Cook JA, Botello AP, Elders A, et al. Systematic review of the agreement of tonometers with Goldmann applanation tonometry. Ophthalmology. 2012;119:1552–7. doi: 10.1016/j.ophtha.2012.02.030 22578443

[31] Kotecha A, Elkarmouty A, Ajtony C, et al. Interobserver agreement using Goldmann applanation tonometry and dynamic contour tonometry: comparing ophthalmologists, nurses and technicians. Br J Ophthalmol. 2016;100:854–9. doi: 10.1136/bjophthalmol-2015-307219 26420825

[32] Garcia-Resua C, Giraldez Fernandez MJ, Yebra-Pimentel E, et al. Clinical evaluation of the Canon TX-10 noncontact tonometer in healthy eyes. Eur J Ophthalmol. 2010;20:523–30. doi: 10.1177/112067211002000326 20037897

[33] Eckert KA, Lansingh VC, McLeod-Omawale J, Furtado JM, Martinez-Castro F, Carter MJ. Field Testing Project to Pilot World Health Organization Eye Health Indicators in Latin America. Ophthalmic Epidemiol. 2018;25: 91–104. doi: 10.1080/09286586.2017.1359848 28945466

[34] Maa AY, Wojciechowski B, Hunt KJ, et al. Early experience with Technology-Based Eye Care Services (TECS): a novel ophthalmologic telemedicine initiative. Ophthalmology. 2017;124:539–46. doi: 10.1016/j.ophtha.2016.11.037 28081944

[35] Kortuem K, Fasler K, Charnley A, et al. Implementation of medical retina virtual clinics in a tertiary eye care referral centre. Br J Ophthalmol. 2018;102:1391–5. doi: 10.1136/bjophthalmol-2017-311494 29306863

[36] Kotecha A, Elkarmouty A, Ajtony C, et al. Interobserver agreement using Goldmann applanation tonometry and dynamic contour tonometry: comparing ophthalmologists, nurses and technicians. Br J Ophthalmol. 2016;100:854–9. doi: 10.1136/bjophthalmol-2015-307219 26420825

[37] Gupta SC, Sinha SK, Dagar AB. Evaluation of the effectiveness of diagnostic & management decision by teleophthalmology using indigenous equipment in comparison with in-clinic assessment of patients. Indian J Med Res. 2013;138: 531–535. 24434260 PMC3868066

[38] Conlin PR, Asefzadeh B, Pasquale LR, Selvin G, Lamkin R, Cavallerano AA. Accuracy of a technology-assisted eye exam in evaluation of referable diabetic retinopathy and concomitant ocular diseases. Br J Ophthalmol. 2015;99: 1622–1627. doi: 10.1136/bjophthalmol-2014-306536 25995299

[39] Maa AY, Medert CM, Lu X, Janjua R, Howell A V., Hunt KJ, et al. Diagnostic Accuracy of Technology-based Eye Care Services: The Technology-based Eye Care Services Compare Trial Part I. Ophthalmology. 2020;127: 38–44. doi: 10.1016/j.ophtha.2019.07.026 31522900

