# Supplementary figures and images for: Teaming-up nurses with ophthalmologists to expand the reach of eye care in a middle-income country: Validation of health data acquisition by nursing staff in a telemedicine strategy
Source: PLoS One. 2021 Nov 30;16(11):e0260594. doi: 10.1371/journal.pone.0260594 (PMC8631620; doi:10.1371/journal.pone.0260594)

A

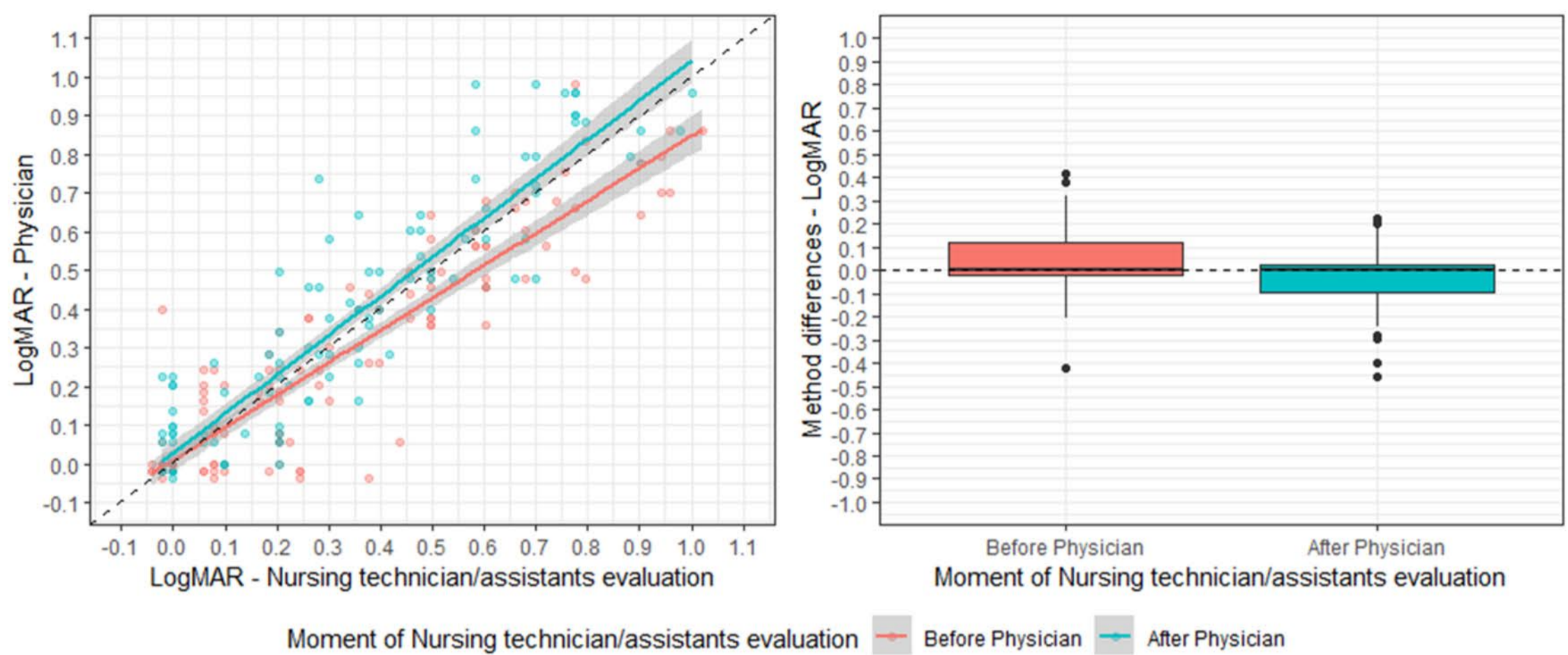

B

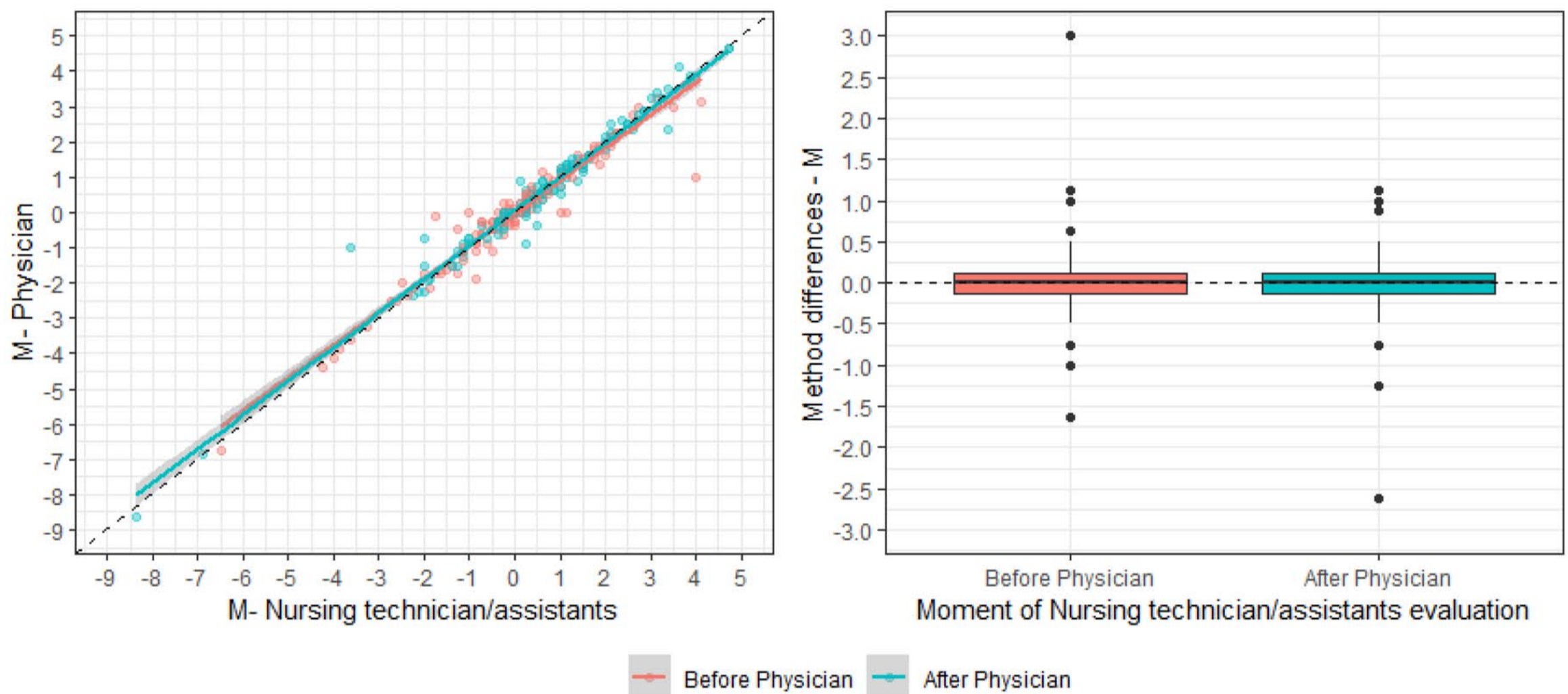

C

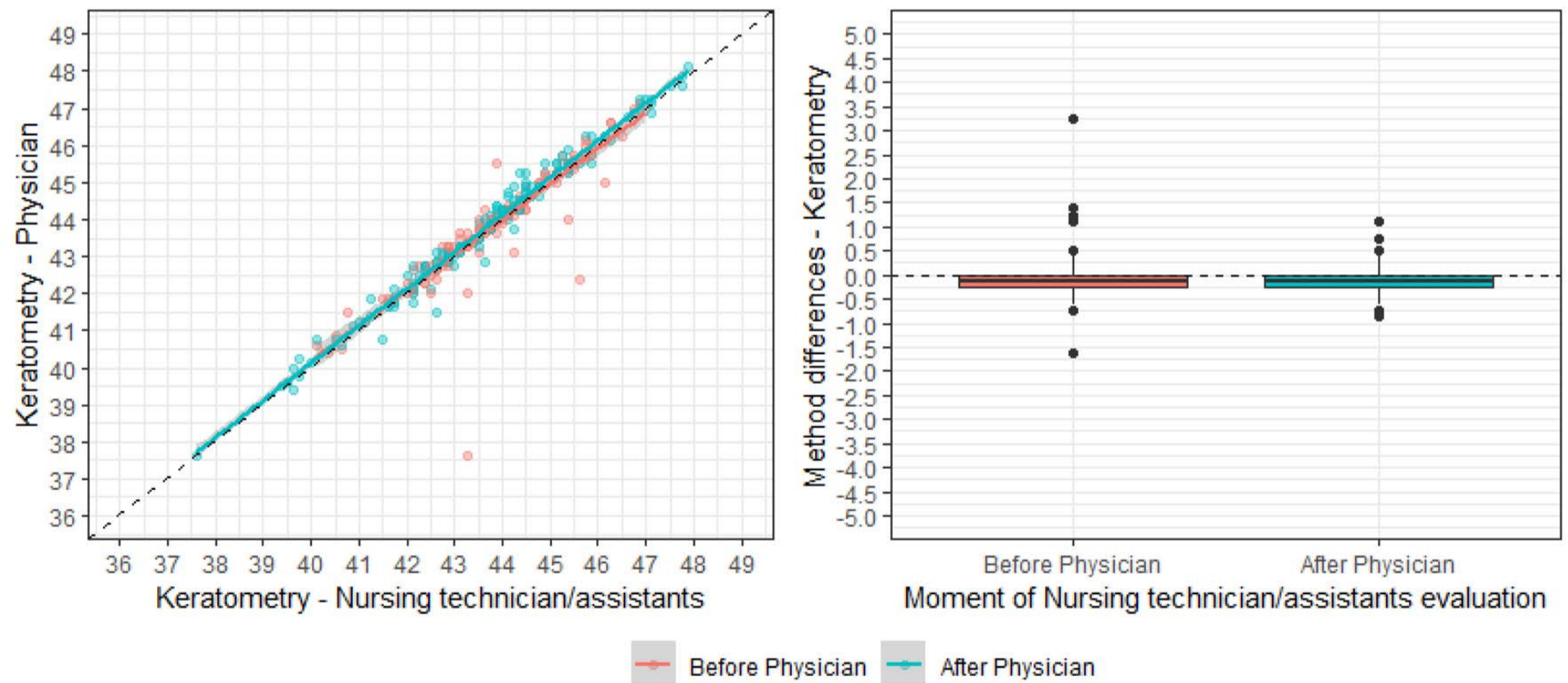

D

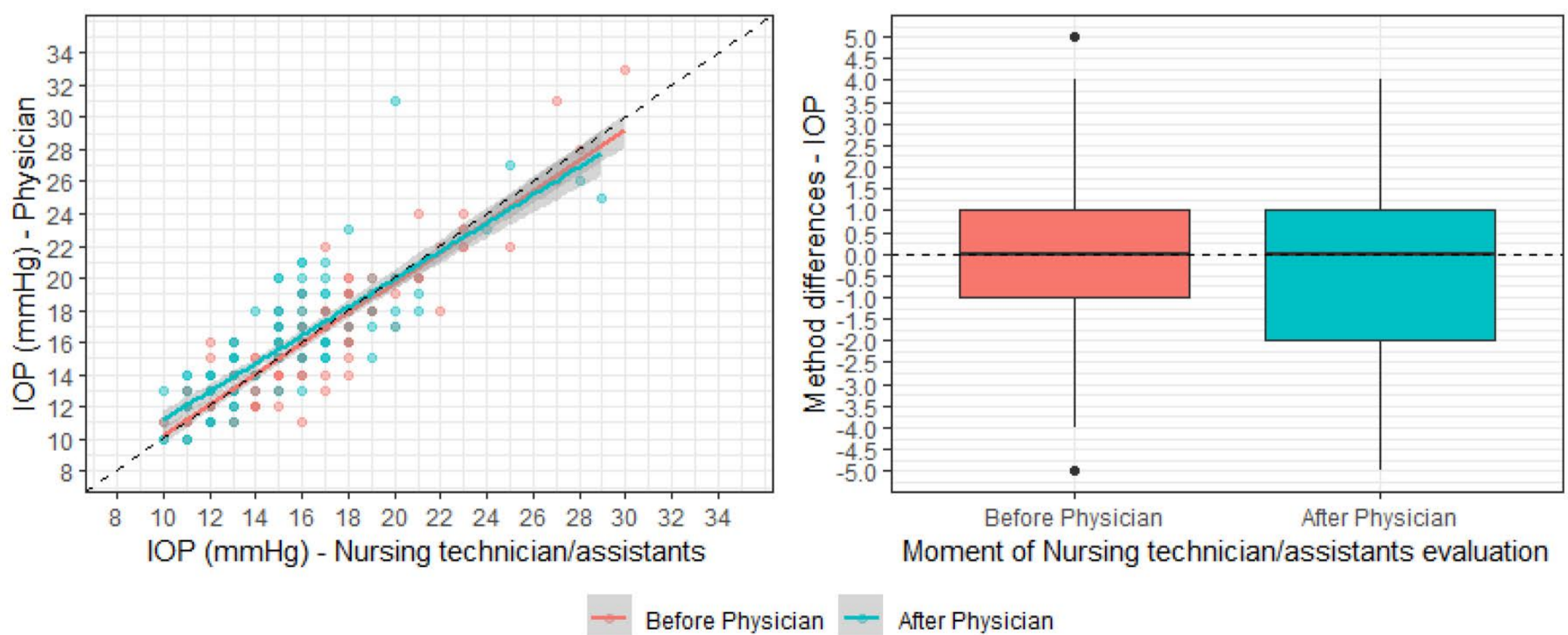

Supplement: S1 Fig — Graphs illustrating correlation between assessments by nursing technicians and doctors: A. Visual acuity, in logMAR; B. Spherical equivalent M, for refraction in diopters; C. Keratometry, in diopters; D. Intraocular pressure, in mmHg. (PDF) [file pone.0260594.s001.pdf]

A

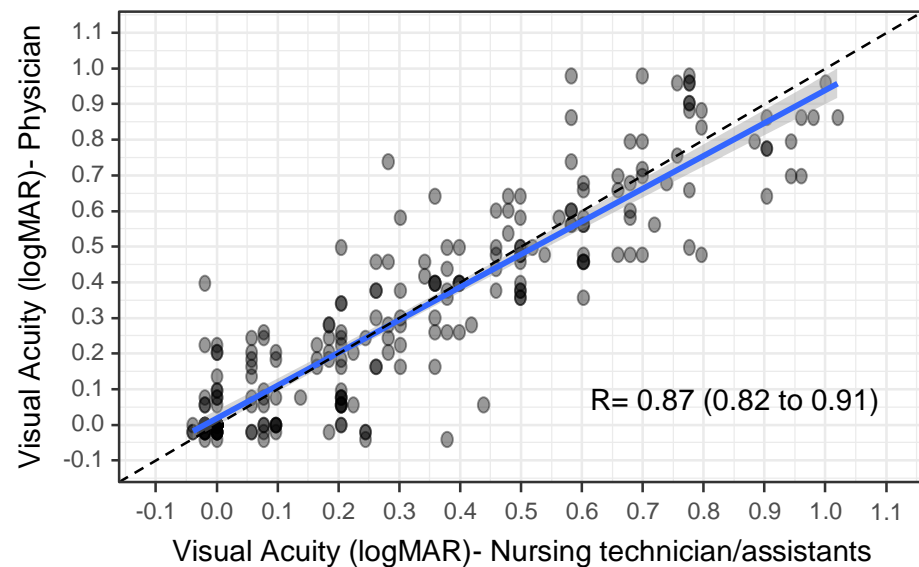

B

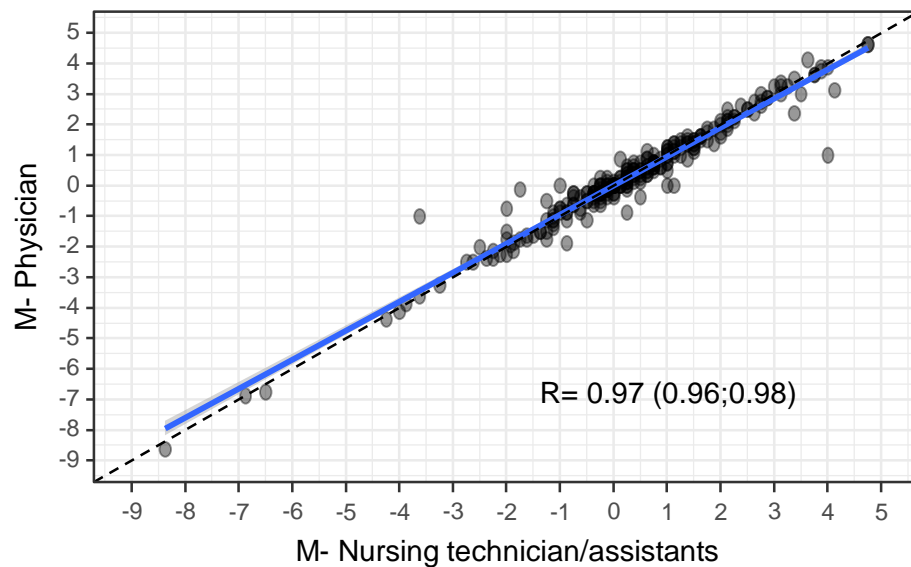

C

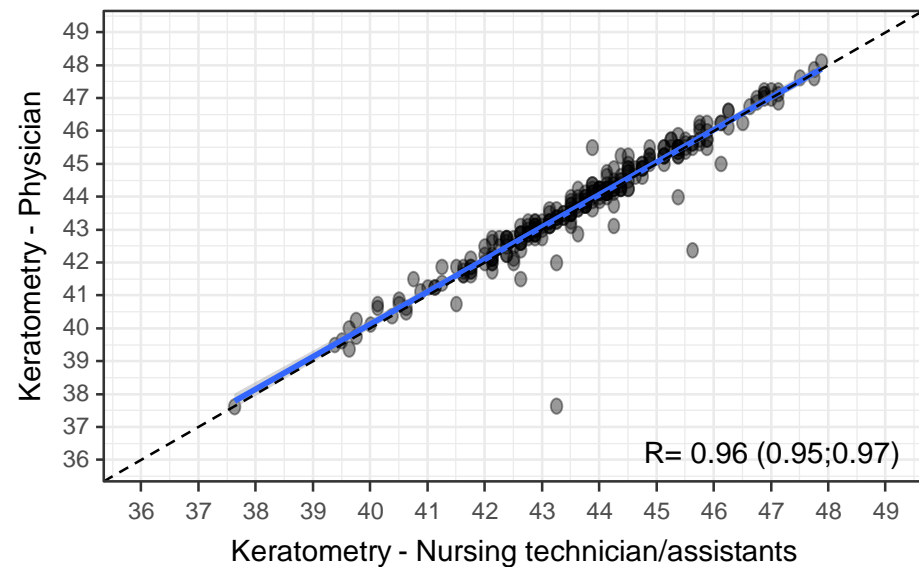

D

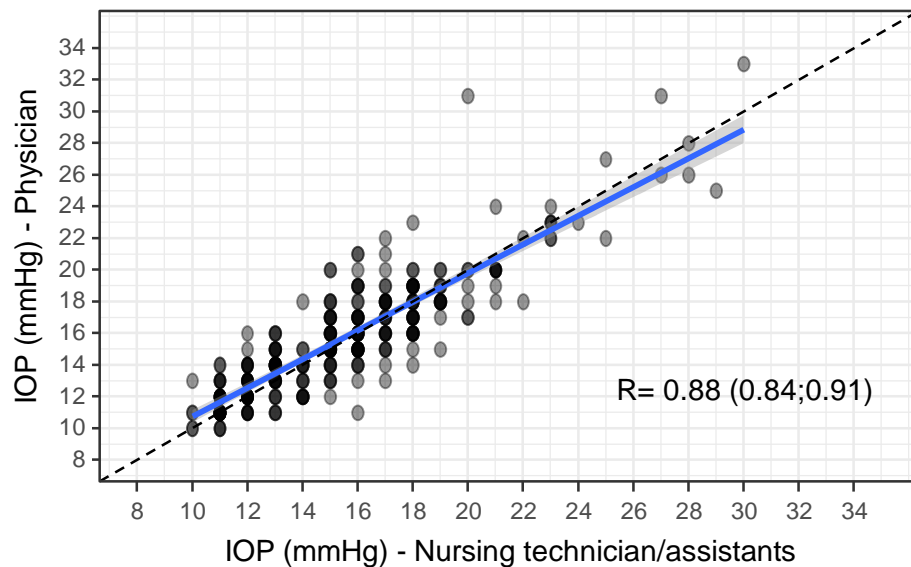

Supplement: S2 Fig — Graph illustrating correlation and discrepancies between measures by order of examination: A) Visual acuity; B) Spherical equivalent M; C) Keratometry; D) Intraocular pressure. (PDF) [file pone.0260594.s002.pdf]

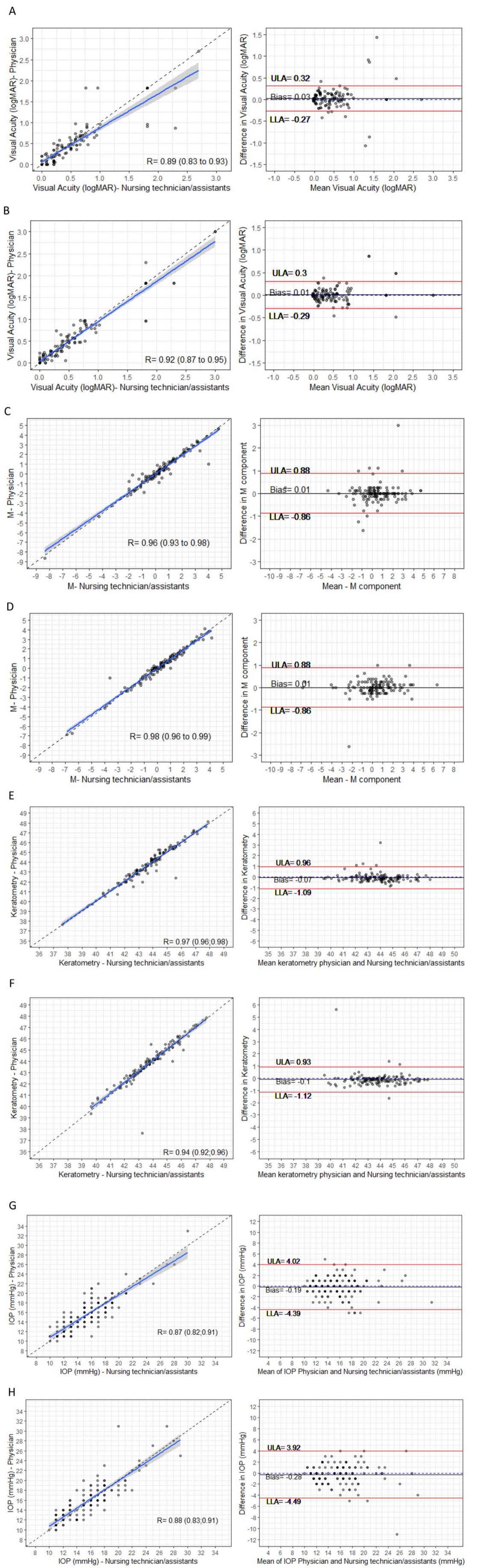

Supplement: S3 Fig — Assessment correlation graphs and Bland-Altman plots comparing assessments by doctors and nursing technicians for: A) Visual acuity for right eye; B) Visual acuity for left eye; C) Spherical equivalent M for right eye; D) Spherical equivalent M for left eye; E) Keratometry for right eye; F) Keratometry for left eye. G) Intraocular pressure for right eye; H) Intraocular pressure for left eye. (PDF) [file pone.0260594.s003.pdf]
